# Chronic Treatment with Ang-(1-7) Reverses Abnormal Reactivity in the Corpus Cavernosum and Normalizes Diabetes-Induced Changes in the Protein Levels of ACE, ACE2, ROCK1, ROCK2 and Omega-Hydroxylase in a Rat Model of Type 1 Diabetes

**DOI:** 10.1155/2014/142154

**Published:** 2014-09-16

**Authors:** Mariam H. M. Yousif, Batoul Makki, Ahmed Z. El-Hashim, Saghir Akhtar, Ibrahim F. Benter

**Affiliations:** ^1^Department of Pharmacology and Toxicology, Faculty of Medicine, Kuwait University, P.O. Box 24923, 13110 Safat, Kuwait; ^2^Department of Pharmacology and Therapeutics, Faculty of Pharmacy, Kuwait University, P.O. Box 24923, 13110 Safat, Kuwait

## Abstract

Angiotensin-(1-7) [Ang-(1-7)] may have beneficial effects in diabetes mellitus-induced erectile dysfunction (DMIED) but its molecular actions in the diabetic corpus cavernosum (CC) are not known. We characterized the effects of diabetes and/or chronic in vivo administration of Ang-(1-7) on vascular reactivity in the rat corpus cavernosum (CC) and on protein expression levels of potential downstream effectors of the renin-angiotensin-aldosterone system (RAAS) such as angiotensin-converting enzyme (ACE), ACE2, Rho kinases 1 and 2 (ROCK1 and ROCK2), and omega-hydroxylase, the cytochrome-P450 enzyme that metabolizes arachidonic acid to form the vasoconstrictor, 20-hydroxyeicosatetraenoic acid. Streptozotocin-treated rats were chronicically administered Ang-(1-7) with or without A779, a Mas receptor antagonist, during weeks 4 to 6 of diabetes. Ang-(1-7) reversed diabetes-induced abnormal reactivity to vasoactive agents (endothelin-1, phenylepherine, and carbachol) in the CC without correcting hyperglycemia. Six weeks of diabetes led to elevated ACE, ROCK1, ROCK 2, and omega-hydroxylase and a concomitant decrease in ACE2 protein expression levels that were normalized by Ang-(1-7) treatment but not upon coadministration of A779. These data are supportive of the notion that the beneficial effects of Ang-(1-7) in DMIED involve counterregulation of diabetes-induced changes in ACE, ACE2, Rho kinases, and omega-hydroxylase proteins in the diabetic CC via a Mas receptor-dependent mechanism.

## 1. Introduction

Erectile dysfunction (ED), a measure of sexual dysfunction or impotency in males, is defined as the inability to achieve and/or maintain an erection sufficient to permit satisfactory sexual intercourse. It is commonly associated with diabetes mellitus (DM) with up to 75% of men with diabetes exhibiting some degree of erectile dysfunction (ED) [1–4]. The incidence of ED, in large part also due to the predicted increase in DM [5, 6], will rise to about 300 million sufferers worldwide by 2025 [7, 8] and represents a significant health burden.

DM-induced ED (DMIED) is multifactorial in aetiology comprising both central (neurogenic) and peripheral (vasculogenic) components and appears more severe and more resistant to treatment compared with nondiabetic ED [9, 10]. For example, treatment with phosphodiesterase (PDE) inhibitors such as sildenafil (Viagra) is not always effective in DMIED for reasons that are not entirely clear [3, 7]. Thus, there is a need for newer more effective therapies based on an increased understanding of the underlying mechanisms of DMIED.

The exact molecular mechanisms by which DM induces ED are not fully known but chronic hyperglycemia likely degrades both neural and vascular endothelium penile control systems that eventually leads to a failure in the neuronal response and/or increase in tone and/or contractility of the smooth muscle within the corpus cavernosum (CC) and penile arteries [7, 9]. Experimental evidence suggests that this may occur via hyperglycemia-induced modulation of nitric oxide (NO) signaling and/or proinflammatory cell signaling pathways and/or elevation in oxidative stress via several pathways including increased glycolysis, polyol pathway flux, formation of advanced glycation, and lipoxygenation end-products [4, 11, 12]. Additionally, there is now a growing body of evidence from our laboratory and others [7, 12–14] on the existence of a local renin-angiotensin-aldosterone system (RAAS) in the penis that plays a critical role in erectile function.

Angiotensin II (Ang II), a major effector of the RAAS, is formed from the actions of angiotensin-converting enzyme (ACE) on Angiotensin 1. It is expressed in the corpus cavernosum and via its AT_1_ receptor activates signaling pathways leading to vasoconstriction, proliferation, fibrosis, and oxidative stress that are thought to play a detrimental role in the progression of DMIED [7, 11–13, 15]. For example, we recently reported in a rat model of type 1 diabetes that Ang II-mediated elevation in oxidative stress, along with a concomitant decrease in antioxidant levels and increased DNA damage, resulted in major cellular degeneration with the diabetic CC that could be blocked either by preventing the formation of Ang II with an ACE inhibitor or by blocking its effects with an AT_1_ receptor antagonist [12].

In contrast to the detrimental ACE/Ang II/AT_1_ receptor “branch” of the RAAS, there also now appears to be a counterregulating or opposing beneficial “branch” that comprises the angiotensin-converting enzyme 2 (ACE2) that can form the heptapeptide, angiotensin-(1-7) (Ang-(1-7)) from Ang II, which mediates its effects via the G-protein coupled receptor known as Mas. The ACE2/Ang-(1-7)/Mas receptor pathway is known to oppose the detrimental effects of ACE/Ang II/AT_1_ receptor in diabetes-induced cardiovascular complications [16–18] and recent evidence suggests it may also be involved in DMIED [7, 12–14, 19]. Several studies have now suggested that Ang-(1-7) has proerectile functions that include enhancing NO-mediated vasodilation, inhibiting penile fibrosis, and attenuating oxidative-stress mediated tissue degeneration [7, 12–14, 20, 21]. For example, we have shown that Ang-(1-7) treatment opposed Ang II-induced oxidative stress and DNA damage that led to penile tissue degeneration in a rat model of type 1 diabetes [12], whereas acute, ex vivo administration of Ang-(1-7) to diabetic rabbit CC segments was effective in attenuating diabetes or Ang II-induced hyperreactivity suggesting a possible role for Ang-(1-7) in the treatment of DMIED [14]. Although these studies suggested that alterations in the fine balance between the ACE/Ang II/AT_1_ receptor and the ACE2/Ang-(1-7)/Mas receptor pathways will likely be important in the development of DMIED, the exact molecular mechanisms underlying the disease and how Ang-(1-7) treatment may impact on specific effector molecules is not known in detail.

## 2. Aims

The aim of this study was to characterize the effects of diabetes and/or chronic administration of Ang-(1-7) on vascular reactivity in the rat CC and in the presence or absence of a MAS receptor antagonist on protein expression levels in the rat CC of (a) ACE1 and ACE2, major components of the two counterregulatory branches of the RAAS; (b) Rho kinases (ROCK) 1 and 2, important contractile regulatory proteins known to be important mediators of ED [22, 23]; and (c) the cytochrome P450 4A1 enzyme, omega-hydroxylase, that metabolizes arachidonic acid (AA) to 20-hydroxyeicosatetraenoic acid (20-HETE), a potent vasoconstrictor [24–26] that we previously identified as a potential target in the treatment of DMIED [19]. Both ROCKs and omega-hydroxylase are known downstream effectors of Ang II in the cardiovascular system [24, 25, 27, 28] but the effect of Ang-(1-7) treatment on the expression of these proteins in the diabetic CC is not known and therefore a subject of this study.

## 3. Methods

### 3.1. Induction of Diabetes

Experiments were performed on male Wistar rats weighing approximately 300 g. The rats had free access to food and water throughout the study. Diabetes was induced by a single ip injection (55 mg/kg, body weight) of streptozotocin (STZ). Rats with fasting blood glucose levels above 250 mg/dL were considered diabetic and included in the study. The animals were sacrificed six weeks after inducing diabetes. Drug treatment was administered for the last 3 weeks prior to sacrificing the animals at 6 weeks.

### 3.2. Animal Groups

All studies involving animals were conducted in accordance with the National Institutes of Health Guide for the Care and Use of Laboratory Animals (NIH Publication number 85-23, Revised in 1985) as approved by Kuwait University Research Administration. Animals included in this study were divided into the following groups (*N* = 12/group): Group 1: nondiabetic (control) animals, Group 2: STZ-treated diabetic animals, Group 3: STZ + Ang-(1-7), and Group 4: STZ + Ang-(1-7) + A779 (a Mas receptor antagonist). Ang-(1-7) was given at a dose of 576 *μ*g/kg/day intraperitoneally (ip) and A779 at a dose of 744 *μ*g/kg/day ip based on our previous studies in animal models [12, 18, 29–33].

### 3.3. Vascular Reactivity Experiments

Rats were anesthetized with ketamine and then sacrificed and the penis was removed en bloc. A ventral incision was made and the cavernosal tissue exposed. The corpus cavernosum was cleaned of the adjacent tissue and cut into longitudinal strips of 2 × 10 mm. Strips of the corpus cavernosum were suspended longitudinally in organ-bath chambers containing 25 mL Krebs Henseleit (KH) solution at pH 7.4, for measurement of isometric tension [14, 19]. The composition of KH-solution was as follows (mM): NaCl (118.3), KCl (4.7), CaCl_2_ (2.5), MgSO_4_ (1.2), NaHCO_3_ (25), KH_2_PO_4_ (1.2), and glucose (11.2). The tissue bath solution was maintained at 37°C and was aerated with 95% oxygen and 5% carbon dioxide mixture. Reactivity of the isolated strips of the corpus cavernosum was determined by measurement of changes in the isometric tension to vasoactive agonists using computerized automatic organ bath LSI Letica Scientific Instruments (Powerlab/8sp ADInstruments, Panlab, Spain) [14, 19]. The preparations were left for 45 min with changing KH solution at 15 min intervals. A pretension of 1.0 gm was applied and the preparations were allowed to stabilize (45 min) until a stable baseline tone was obtained.

### 3.4. Effect of Vasoactive Agonists

Cumulative concentration response curves were established to investigate the contractile responses of the corpus cavernosum to the constrictor agonists phenylephrine (PE) and endothelin-1 (ET-1). Ascending concentrations of the agonists PE (10^−9^–5 × 10^−3^ M) and ET-1 (10^−10^–10^−6^ M) were applied to establish a cumulative dose response curve using corpus cavernosal strips isolated from the different animal groups. The response to any given concentration of the agonists was left to stabilize before adding the next drug concentration. In another set of experiments, the relaxant responses to carbachol were investigated in the cavernosal strips from the different animal groups. The relaxant response to the agonists were determined after precontracting the tissues with submaximal dose of PE (3 × 10^−7^ M) added to the organ baths. After obtaining a steady level of precontraction, the relaxant effects to carbachol (10^−9^–10^−5^ M) were tested on different preparations of the corpus cavernosum. The response to each concentration of the agonists was left to stabilize before adding the next drug concentration. The relaxant responses were expressed as percentage reduction of the tension induced by precontraction with PE.

### 3.5. Western Blotting Studies

Western blotting for ACE (band detected at molecular weight of approximately 195 kDa), ACE2 (90–100 kDa), ROCK1 and ROCK2 (both at around 160 kDa), and omega-hydroxylase (58 kDa) was performed similar to that described by us previously [32, 34]. Strips of the corpus cavernosum were dissected from the different animal groups, cut into small pieces, and homogenized in appropriate amounts of lysis buffer (pH 7.6) containing 50 mM Tris-base, 5 mM EGTA, 150 mM NaCl, 1% Triton 100, 2 mM Na_3_VO_4_, 50 mM NAF, 1 mM PMSF, 20 *μ*M phenylarsine, 10 mM sodium molybdate, 10 *μ*g/mL leupeptin, and 8 *μ*g/mL aprotinin. The tissue was homogenized at half maximum speed for 1 minute and then left to lyse completely by incubation on shaking ice-cold shaker for 1 hour. Lysates were centrifuged at 12000 rpm at 4°C for 15 minutes. Supernatants were then collected and protein concentration was estimated using Lowry's method. Then the samples were mixed with sample loading buffer and lysis buffer to make all the samples of the same protein concentration. The samples were then boiled for 5 min and subjected to SDS polyacrylamide gel electrophoresis. The separated proteins were then transferred to PVDF membrane for 1 hour at 95 V. After blocking, membranes were incubated with the primary antibodies specific for each protein overnight and subsequently with appropriate secondary antibodies conjugated to horseradish peroxidase (Amersham, UK). The following antibodies were used in this study: ACE (H-170): sc-20791, ACE2 (H-175): sc-20998, ROCK1 (H-85): sc-5560, ROCK2 (H-85): sc-5561 (Santa Cruz, USA), and omega-hydroxylase (cytochrome P450 4A1): ab22615 (Abcam, USA). In addition, the anti-*β*-actin rabbit polyclonal IgG (1 *μ*L/10 mL) Cat. number A-2066 was obtained from Sigma Chemical Co., USA. Immunoreactive bands were detected with SuperSignal Chemiluminescent Substrate (Pierce, UK) using Kodak autoradiography film (G.R.I., Rayne, UK). To ensure equal loading of proteins *β*-actin levels were detected using primary rabbit anti-human *β*-actin antibody followed by the secondary anti-rabbit IgG horseradish peroxidase conjugated antibody (Cell Signaling, USA). Images were finally analysed and quantified by densitometry and all data normalized to *β*-actin levels as described by us previously [32, 34].

### 3.6. Statistical Analysis

Results were analyzed using GraphPad Prism software. Data are presented as Mean ± SE of “*n*” number of experiments. Mean values were compared using either 2-way analysis of variance or analysis of variance followed by post hoc test (Bonferroni) as appropriate. The difference was considered to be significant when *P* value was less than 0.05.

#### 3.6.1. Main Outcome Measures

Vascular responsiveness of isolated CC strips to vasoactive agents (endothelin-1, phenylephrine, and carbachol) and protein levels were determined by western blotting.

## 4. Results

### 4.1. Blood Glucose Levels and Body Weights

STZ-induced diabetes led to a significant elevation in blood glucose concentration. Hyperglycemia persisted in the diabetic animals and was 31.2 ± 1.7 mmol·L^−1^ after 6 weeks of diabetes as compared with 4.7 ± 0.7 mmol·L^−1^ in the nondiabetic control animals but 3-week chronic treatment with Ang-(1-7) (30.8 ± 1.4 mmol·L^−1^) did not significantly reduce blood glucose levels. There was a significant reduction in the weights of STZ-diabetic rats (145 ± 8 g) compared with the nondiabetic control animals (219 ± 5 g) after 6 weeks of diabetes, whereas Ang-(1-7) treatment significantly improved the weight of diabetic rats to 188 ± 11 g.

### 4.2. Chronic Treatment with Ang-(1-7) Reverses Diabetes-Induced Abnormal Vascular Reactivity in the Rat CC

**Figure 1:**
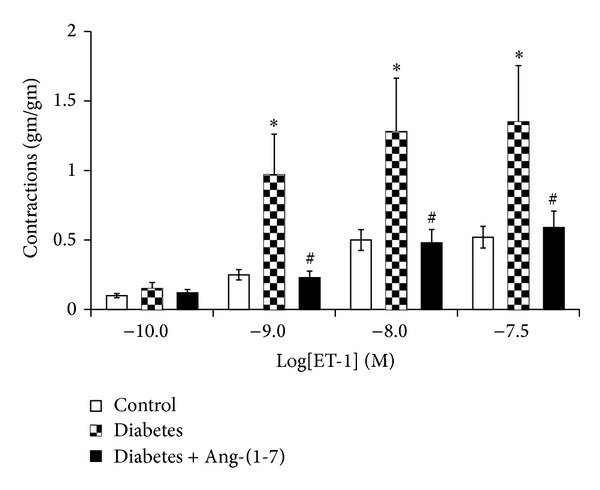
Endothelin-1-induced vasoconstriction in the corpus cavernosum segments from nondiabetic (control), diabetic, and diabetic rats treated with Ang-(1-7) (Mean ± SEM, *n* = 6–8). Asterisk (*) indicates significantly different mean values as compared to control, and hash (#) indicates significantly different mean values as compared to diabetes. *P* < 0.05. Tissues isolated from 6-week diabetic rats treated with Ang-(1-7) for the last 3 weeks of the study.

**Figure 2:**
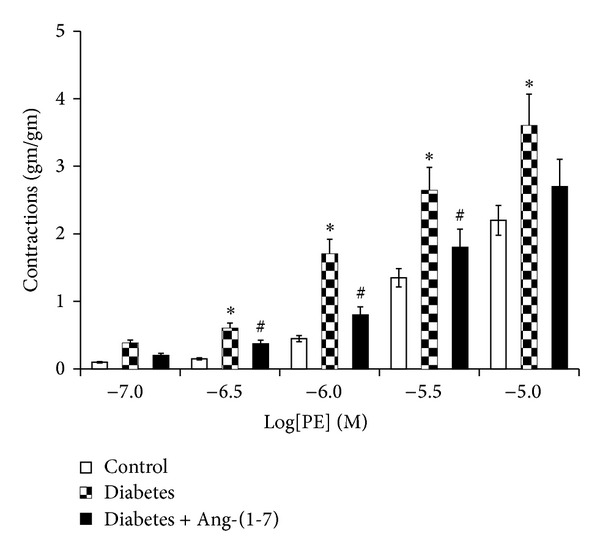
Phenylephrine-induced vasoconstriction in the corpus cavernosum segments from nondiabetic (control), diabetic, and diabetic rats treated with Ang-(1-7) (Mean ± SEM, *n* = 6–8). Asterisk (*) indicates significantly different mean values as compared to control, and hash (#) indicates significantly different mean values as compared to diabetes. *P* < 0.05. Tissues isolated from 6-week diabetic rats treated with Ang-(1-7) for the last three weeks of the study.

**Figure 3:**
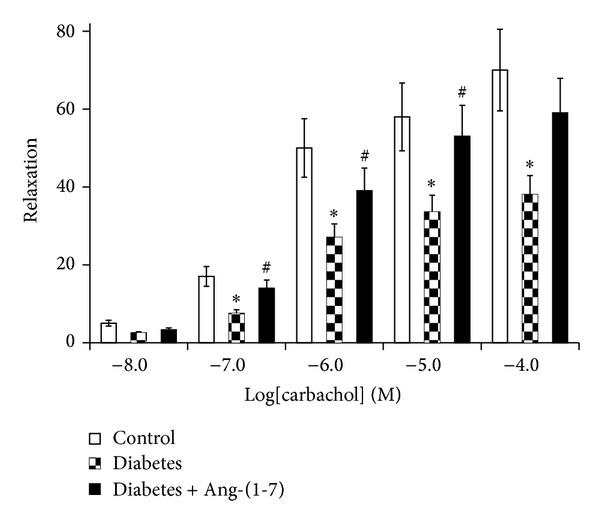
Carbachol-induced relaxation (expressed as %) in the corpus cavernosum segments from nondiabetic (control), diabetic, and diabetic rats treated with Ang-(1-7) (Mean ± SEM, *n* = 6–8). Asterisk (*) indicates significantly different mean values as compared to control, and hash (#) indicates significantly different mean values as compared to diabetes. *P* < 0.05. Tissues isolated from 6-week diabetic rats treated with Ang-(1-7) for the last three weeks of the study.

Figures 1 and 2 show that PE and ET-1, respectively, produced a concentration-dependent contraction, while carbachol (Figure 3) produced relaxation in the isolated CC from both nondiabetic control and diabetic rats. In the CC tissue strips from diabetic animals, the constrictor responses to ET-1 and PE were significantly potentiated, whereas the relaxant responses to carbachol were significantly attenuated (*P* < 0.05). However, diabetes-induced abnormal reactivity to the vasoactive agonists was significantly corrected in cavernosal tissues isolated from diabetic animals chronically treated with Ang-(1-7) (*P* < 0.05) (Figures 1–3).

### 4.3. Chronic Treatment with Ang-(1-7) Attenuates Diabetes-Induced Changes in ACE and ACE2 Protein Expression via Its Mas Receptor

**Figure 4:**
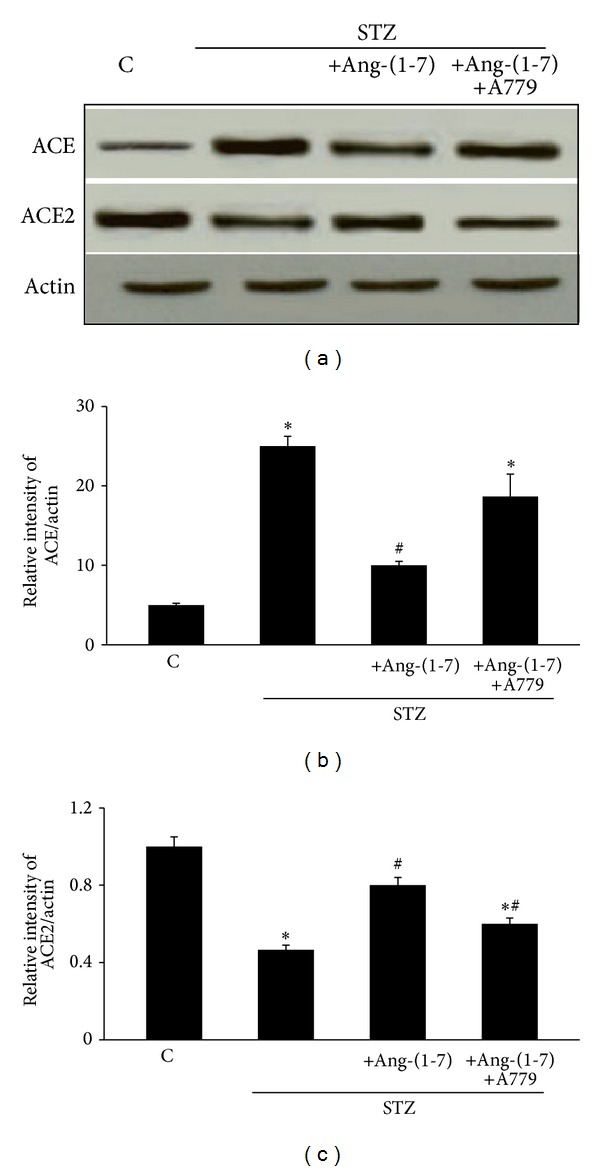
The levels of ACE and ACE2 protein expression in corpus cavernosum of nondiabetic (C), diabetic (STZ), and STZ-rats chronically treated either with Ang-(1-7) alone (STZ + Ang-(1-7)) or in combination with A779 (STZ + Ang-(1-7) + A779). Panel (a) is a representative western blot showing the levels of ACE, ACE2, and the protein loading control, *β*-actin. Panels (b) and (c) are densitometry histograms showing levels of ACE and ACE2 normalized to actin, respectively. *N* = 4; Mean ± SD. Asterisk (∗) indicates significantly different (*P* < 0.05) mean values from normal nondiabetic rats (C), whereas hash (#) indicates significantly different mean values (*P* < 0.05) from diabetic rats (STZ).

Figure 4 shows that 6 weeks of diabetes led to significantly increased expression of ACE and decreased expression of ACE2 proteins in the rat CC. Chronic treatment with Ang-(1-7) significantly attenuated diabetes-induced changes in both proteins (*P* < 0.05). Chronic coadministration of the Mas receptor antagonist, A779, with Ang-(1-7) reversed the effects of Ang-(1-7) on ACE and ACE protein expression in the diabetic rat CC (*P* < 0.05).

### 4.4. Chronic Ang-(1-7) Treatment Corrects Diabetes-Induced Elevation in ROCK1 and ROCK2 Protein Expression via Its Mas Receptor

**Figure 5:**
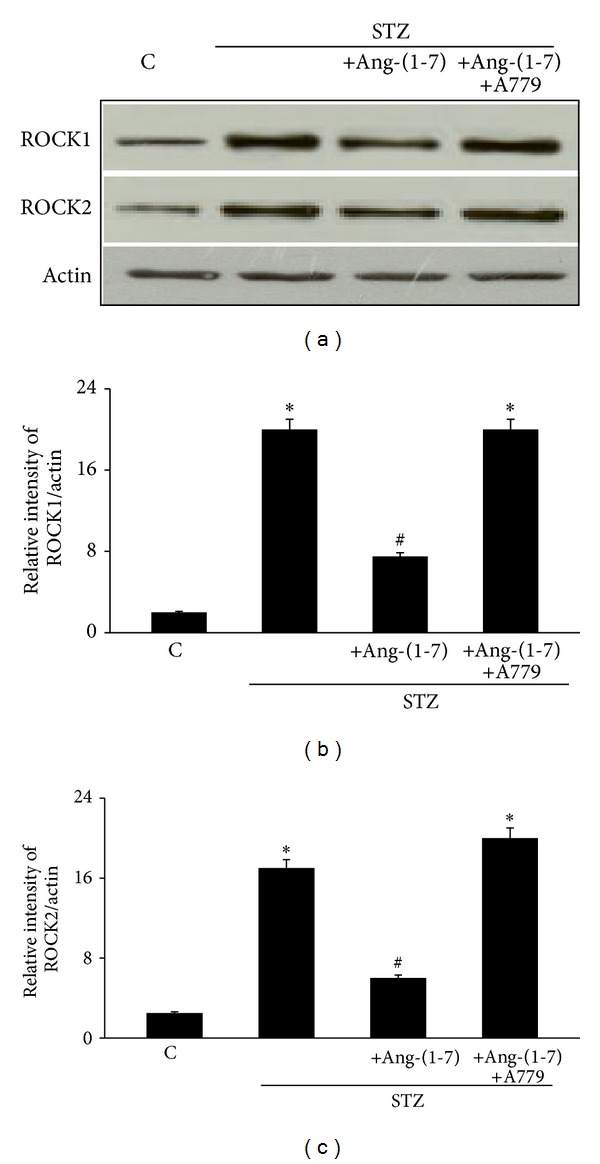
The levels of ROCK1 and ROCK2 protein expression in corpus cavernosum of nondiabetic (C), diabetic (STZ), and STZ-rats chronically treated either with Ang-(1-7) alone (STZ + Ang-(1-7)) or in combination with A779 (STZ + Ang-(1-7) + A779). Panel (a) is a representative western blot showing the levels of ROCK1, ROCK2, and the protein loading control, *β*-actin. Panels (b) and (c) are densitometry histograms showing levels of ROCK1 and ROCK2 normalized to actin, respectively. *N* = 4; Mean ± SD. Asterisk (∗) indicates significantly different (*P* < 0.05) mean values from normal nondiabetic rats (C), whereas hash (#) indicates significantly different mean values (*P* < 0.05) from diabetic rats (STZ).

Figure 5 shows that diabetes led to significantly increased protein expression of ROCK1 and ROCK2 in the rat CC (*P* < 0.05). Chronic treatment with Ang-(1-7) significantly attenuated diabetes-induced changes in both ROCK proteins (*P* < 0.05). Chronic coadministration of A779 with Ang-(1-7) reversed the effects of Ang-(1-7) on ROCK1 and ROCK2 protein expression in the diabetic rat CC (*P* < 0.05) to a level that was not significantly different to nondiabetic controls (Figure 5).

### 4.5. Chronic Treatment with Ang-(1-7) Attenuates Diabetes-Induced Increase in Omega-Hydroxylase Protein Expression via Its Mas Receptor

**Figure 6:**
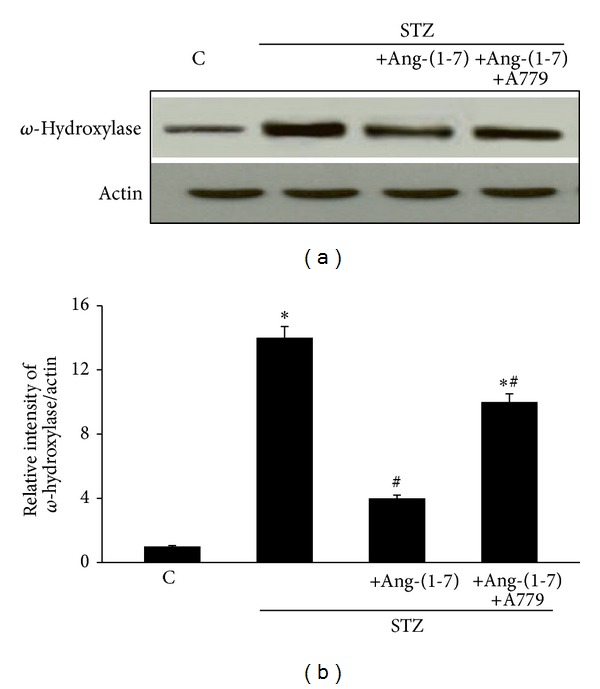
The level of omega-hydroxylase protein expression in the corpus cavernosum of nondiabetic (C), diabetic (STZ), and STZ-rats chronically treated either with Ang-(1-7) alone (STZ + Ang-(1-7)) or in combination with A779 (STZ + Ang-(1-7) + A779). Panel (a) is a representative western blot showing the level of omega(*ω*)-hydroxylase protein and *β*-actin as a loading control. Panels (b) and (C) are densitometry histograms showing levels of omega(*ω*)-hydroxylase protein normalized to actin. *N* = 4; Mean ± SD. Asterisk (∗) indicates significantly different (*P* < 0.05) mean values from normal nondiabetic rats (C), whereas hash (#) indicates significantly different mean values (*P* < 0.05) from diabetic rats (STZ).

Figure 6 shows that diabetes led to significantly increased expression of omega-hydroxylase protein in the rat CC (*P* < 0.05). Chronic treatment with Ang-(1-7) significantly attenuated diabetes-induced changes in omega-hydroxylase protein expression (*P* < 0.05). Chronic coadministration of A779 with Ang-(1-7) significantly attenuated the effects of Ang-(1-7) on omega-hydroxylase protein expression in the diabetic rat CC (*P* < 0.05) (Figure 5).

## 5. Discussion

The major findings of this study were that diabetes led to an upregulation in ACE, ROCK1, ROCK2, and omega-hydroxylase proteins and a downregulation in ACE2 protein that was accompanied by abnormal vascular reactivity in the rat CC. Importantly, chronic treatment with exogenous Ang-(1-7) was able to reverse diabetes-induced abnormal CC vascular reactivity without correcting hyperglycemia and via its Mas receptor normalized diabetes-induced changes in ACE, ACE2, ROCK1, ROCK2, and omega-hydroxylase proteins in the rat CC. Our study, therefore, showed for the first time that an imbalance in the ACE-ACE2 enzymes is associated with development of DMIED and further highlights ROCKs and omega-hydroxylase as novel protein effectors by which Ang-(1-7) mediates its beneficial effects in DMIED. Furthermore, since only a relatively short term, 3-week chronic treatment with Ang-(1-7) in animals with preestablished diabetes was effective in reversing DM-induced protein and vascular reactivity changes in the diabetic CC, our study has clinical relevance in suggesting that therapeutic strategies aimed at activating the endogenous ACE2/Ang-(1-7)/Mas receptor signaling pathway for even a short time course may be beneficial in DMIED.

The fact that in our study we were able to measure detectable levels of ACE and ACE2 in normal and diabetic CC provided further evidence for the existence of a local renin-angiotensin system (RAS) within the CC [3, 7, 11]. Moreover, since diabetes led to a significant upregulation of ACE and downregulation of ACE2 in the rat CC (Figure 4), our data implies that disruption in the ACE-ACE2 balance with a resulting hyperactivity of the detrimental ACE/Ang II/AT_1_ receptor “branch” of the RAAS together with a concomitant attenuation of ACE2/Ang-(1-7)/Mas receptor signaling pathway is associated with the development of DMIED. Our finding in the diabteic rat CC is consistent with the development of other cardiovascular and renal pathologies that are also thought to involve an imbalance in ACE-ACE2 enzymes representing the two opposing “branches” of the RAAS [16, 17, 35].

The observed upregulation of ACE in the diabetic CC (Figure 4), which converts the inactive decapeptide Ang I into the octapeptide Ang II, the major effector of the RAAS, is consistent with previous reports showing higher levels of Ang II in the diabetic CC [36–38] that lead to deleterious effects, such as vasoconstriction, proliferation, fibrosis, and oxidative stress [7, 12, 15]. Pharmacological blockage of Ang II/AT_1_ actions using ACE inhibitors and/or AT_1_ receptor blockers (ARBs) has beneficial effects on erectile function in animal models and humans [1, 3, 11–13]. Since both ACE inhibitors and ARBs can increase Ang-(1-7) levels in plasma and tissue [39], it is possible that the beneficial effects of the Ang II blockade on erectile function might, at least in part, be mediated by Ang-(1-7). Indeed, we previously showed that not only modulation of Ang II signaling by captopril and losartan but also administration of Ang-(1-7) restored the diabetes-induced structural changes and oxidative DNA damage in the diabetic CC [12].

Our observation that ACE2 is downregulated in diabetes suggests reduced formation of endogenous Ang-(1-7) in the diabetic rat CC, though its levels were not measured directly in this study. Importantly, our results showed that chronic 3-week daily treatment with exogenous Ang-(1-7) was able to reverse diabetes-induced changes in ACE1 and ACE2 and vascular reactivity in the rat CC presumably by activation of the endogenous ACE2/Ang-(1-7)/Mas receptor pathway and readdressing of the proposed imbalances in the two counterregulatory branches of the RAAS in the diabetic CC.

The fact that administration of Ang-(1-7) also reversed diabetes-induced upregulation of Rho kinases, ROCK1 and ROCK2, suggests a novel mechanism by which Ang-(1-7) may exerts its beneficial effects on DMIED. Upregulation of the Rho/ROCK pathway has been implicated in a variety of cardiovascular complications including ED [13, 15, 22] and may represent a downstream effector of Ang II. ACE/Ang II/AT_1_ receptor signaling is known to exert its vasoconstrictor effects on smooth muscle via its downstream effects on Rho/ROCK pathway [15, 40, 41]. Thus, ROCK upregulation might be contributing to the observed exaggerated responsiveness of the diabetic rat CC to vasoconstrictor agents (Figures 1 and 2) and attenuated response to relaxant effects of carbachol (Figure 3) in a manner similar to that observed by us in the diabetic mesenteric bed where diabetes-induced altered vascular reactivity was corrected upon ROCK inhibition [34]. Inhibition of ROCKs is also known to improve erectile function by a variety of mechanisms including increased NO signaling and suppressing penile apoptosis and corporal fibrosis [11, 42–44]. As such, ROCK inhibitors are increasingly being considered for the treatment of ED [1, 11]. Rho-kinase inhibitors improve erectile function in hypertensive, diabetic, and aged rats as well as in cavernous nerve injury induced rats [11, 42]. These reports clearly illustrate that direct inhibition of RhoA/Rho-kinase pathway may represent a suitable therapeutic approach in the treatment of ED. However, our data presented here suggests for the first time that chronic treatment with Ang-(1-7) can also reverse upregulation of ROCKs in the diabetic CC and may represent a novel alternative to conventional ROCK inhibitors for the treatment of ED especially in DMIED. Whether attenuation of ROCK protein expression in diabetic CC occurs via a direct effect of Ang-(1-7) or by counterregulation of ACE/Ang II/AT_1_ receptor pathway is not entirely clear and requires further study.

The fact that diabetes led to an elevation in omega-hydroxylase protein expression implies that DMIED is associated with increased production of 20-HETE-a potential downstream effector of the ACE/Ang II/AT_1_ receptor “branch” of the RAAS [24] though its levels were not measured here and is a potential limitation of this study. However, we previously showed that Ang II via its AT_1_ receptors increases 20-hydroxyeicosatetraenoic acid (20-HETE) production in vascular smooth muscle cells [28]. It is now well established that an imbalance in the metabolism of AA and 20-HETE levels contributes to development of cardiovascular dysfunction and end-organ damage [20, 24, 25, 45–48] but this is the first report in the CC showing an imbalance in omega-hydroxylase being associated with DMIED. 20-HETE is a potent vasoconstrictor that has important roles in the regulation of vascular tone in several different tissues [24]. Its upregulation in the diabetic CC implies that it may have a significant role in contributing to the exaggerated vasoconstrictor response to PE and ET-1 observed in the diabetic CC in this study (Figures 1 and 2). Elevated 20-HETE production is also known to occur in other vasculatures as well as the heart, liver, and kidney of diabetic animals [24, 47, 48]. Indeed, we were the first to show that selective inhibition of 20-HETE production with N-hydroxy-N′-(4-butyl-2-methyl-phenyl)-formamidine (HET0016; an inhibitor of CYP450 4A1, omega-hydroxylase) can prevent diabetes-induced hyperreactivity to vasoconstrictors in the rat carotid artery [47] and can further attenuate cardiovascular end-organ damage in animal models of diabetes and/or hypertension [20, 45, 48]. Interestingly, inhibition of 20-HETE also improved vasodilator responses in several vascular beds [20, 45, 48]. Thus, removal of the vasoconstrictor signaling mediated via 20-HETE leads to correction of diabetes-induced abnormal responsiveness to vasoconstrictors and vasodilators. This may also be occurring in our present study in the CC where we observed exaggerated CC vascular reactivity to PE and ET-1 (Figures 1 and 2) and an attenuated responsiveness to the vasorelaxant, carbachol (Figure 3), after 6 weeks of diabetes. Although not directly studied here, we previously showed that acute, ex vivo inhibition of omega-hydroxylase with HET0016 in CC segments resulted in correction of abnormal reactivity to phenylephrine and carbachol in diabetic and aged rats [19]. The fact that in the present study we observed a normalization of the abnormal CC reactivity upon chronic treatment with Ang-(1-7) suggests that Ang-(1-7) can also counterregulate omega-hydroxylase upregulation and the likely increased 20-HETE production in the diabetic CC. Taken together, our data are therefore consistent with the hypothesis that ACE/Ang II/AT_1_ receptor arm of the RAAS and likely downstream effectors, omega-hydroxylase/20-HETE and Rho/ROCK pathways, are driving the vasoconstriction and other deleterious antierectile actions in DMIED, whereas ACE2/Ang-(1-7)/Mas receptor signaling pathway leads to vasorelaxation and generally beneficial or proerectile actions in DMIED.

The data presented here is also consistent with previous reports on the beneficial actions of Ang-(1-7) in erectile function. Both Ang-(1-7) and its Mas receptor are known to be present in the human CC [11, 21]. Our group previously reported that Ang-(1-7) via Mas receptor can produce nitric oxide-dependent relaxation of the rabbit CC [14]. We further showed that acute, ex vivo preincubation of the diabetic rabbit CC with Ang-(1-7) corrected the diabetes-induced hyperresponsiveness to Ang II [14]. Ang-(1-7) has also been shown to normalize the severely depressed erectile function observed in DOCA-salt hypertensive rats by activation of Mas receptor and subsequent NO release [21] and oral delivery of Ang-(1-7) in a cyclodextrin formulation reduced penile fibrosis and improved cavernosal endothelial function in mice with hypercholesterolemia [7]. Additionally, also the synthetic Mas receptor agonist, AVE 0991, potentiated rat penile erectile response [49]. These studies suggest that proerectile vasculogenic actions of the ACE2/Ang-(1-7)/Mas receptor axis involve increased cavernosal vasodilation by increasing NO production/bioavailability [7, 14, 20, 21, 49], reduced oxidative-stress mediated damage to DNA and penile tissue [12], and blocking development of penile fibrosis [7, 13]. Although the above studies and our data presented here suggest that Ang-(1-7) counteracts the ACE/Ang II/AT1 receptor arm of the RAAS in the CC via its Mas receptor, there is a possibility that it may also block AT_1_ and/or stimulate AT_2_ receptors in certain cell types and tissues [7]. However, whether Ang-(1-7), in addition to its effects via Mas receptor, affects AT_1_ or AT_2_ receptors in the penis remains to be studied.

In conclusion, in this study we showed that diabetes-induced abnormal cavernosal vascular reactivity, a marker for DMIED, was accompanied by imbalances in ACE-ACE2, ROCK1, ROCK2, and omega-hydroxylase proteins which could be reversed by 3-week chronic treatment with Ang-(1-7) via activation of its Mas receptor. Whether more longer-term treatments with Ang-(1-7) would have any additional benefit remains to be determined. However, despite the caveat that only protein expression and not activity was measured, our study provides a novel insight into the mechanism of action of ACE2/Ang-(1-7)/Mas receptor pathway in the diabetic CC whereby we show for the first time that the beneficial effects of Ang-(1-7) in DMIED are at least partially due to inhibition of omega-hydroxylase, ROCKs, and ACE levels through activation of its Mas receptor. Thus, we further suggest that activating the endogenous ACE2/Ang-(1-7)/Mas receptor pathway of the RAAS system may represent an attractive strategy for the treatment of ED associated with diabetes.
